# Management of Non-Infectious Uveitis, a Selection of Topical Items Updating

**DOI:** 10.3390/jcm11195558

**Published:** 2022-09-22

**Authors:** Pascal Sève, Thomas El Jammal, Mathieu Gerfaud-Valentin, Laurent Kodjikian, Yvan Jamilloux, Robin Jacquot

**Affiliations:** 1 Department of Internal Medicine, Hôpital de la Croix-Rousse, Université Claude Bernard Lyon 1, F-69100 Lyon, France; 2 Hospices Civils de Lyon, Pôle IMER, F-69003 Lyon, France; 3 HESPER EA 7425, University Claude Bernard-Lyon 1, F-69008 Lyon, France; 4 Department of Ophthalmology, Hôpital de la Croix-Rousse, Université Claude Bernard Lyon 1, F-69100 Lyon, France

First of all, we would like to thank all of the authors for their contributions and the editorial staff who enabled the achievement of this «Diagnosis and Management of Non-infectious Uveitis: Old and New Challenges» Special Issue. This issue covers a selection of hot topics on non-infectious uveitis and the daily clinical practice in its management. Significant progress has been made both in understanding the pathogenesis and in diagnostic and therapeutic approaches, particularly in terms of changes in diagnosis and classification, and a more comprehensive approach to care. Therefore, the issue contains very didactic and practical general reviews, as well as original articles focused on little-known or evolving topics.

With 80 causes, the etiological diagnosis of uveitis is complex and challenging [1]. One way to help junior and general ophthalmologists is to develop an algorithm, which was the objective of Jamilloux and colleagues’ work [2]. The authors implemented and validated a Bayesian belief network algorithm for the differential diagnosis of the most relevant causes of uveitis. The training dataset (*n* = 897) and the test dataset (*n* = 154) were composed of all incident cases of uveitis admitted in two internal medicine departments in two independent French centres. The etiologies of uveitis were classified into eight groups, including idiopathic uveitis, sarcoidosis, spondyloarthritis (Spa) and HLA-B27-associated, tuberculosis, Behcet’s disease (BD), multiple sclerosis, lymphoma, and other inflammatory diseases. The algorithm was based on simple epidemiological characteristics (age, sex, and ethnicity) and anatomoclinical features of uveitis. This Bayesian belief network allowed the identification of uveitis etiology with good performance when the two most likely diagnoses were sought. The estimate obtained by cross-validation in the training dataset concluded that the uveitis etiology determined by experts corresponded to one of the two most likely diagnoses in at least 77% of the cases. By using the estimate derived in the test dataset, this probability reached at least 83%. Such an algorithm could guide diagnostic work-up and assist young physicians in the selection of further diagnostic investigations.

In addition to the routine clinical diagnosis of uveitis, the Standardization of Uveitis Nomenclature (SUN) Working Group recently published classification criteria for 25 uveitis conditions in an issue of the American Journal of Ophthalmology [3]. This monumental, decade-long effort was conducted in four phases: (1) informatics, (2) case collection, (3) case selection, and (4) machine learning. Using formal consensus techniques, a final database was constructed of 4046 cases achieving supermajority agreement on the diagnosis. This database was analysed by anatomical class; cases for each class were randomly separated into a training set (~85% of the cases) and a validation set (~15% of the cases) for each disease. The classification of the 25 uveitis had high overall accuracy (i.e., low misclassification rates) and seems to perform well for use in clinical and translational research. For illustration purposes, the key criteria for sarcoidosis-associated uveitis included a compatible uveitic syndrome of any anatomic class and evidence of sarcoidosis, either (1) tissue biopsy demonstrating non-caseating granulomata or (2) bilateral hilar adenopathy on chest imaging [4]. The overall accuracy of the diagnosis of sarcoidosis-associated uveitis in the validation set was 99.7% (95% confidence interval: 98.8–99.9). The misclassification rates for sarcoidosis-associated uveitis in the training sets were 3.2% in anterior uveitis, 2.6% in intermediate uveitis, and 1.2% in panuveitis; in the validation sets, the misclassification rates were 0% in anterior uveitis, 0% in intermediate uveitis, and 0% in panuveitis. In sum, the classification criteria developed by the SUN working group should be used in clinical and translational research.

Spa and HLA-B27-associated uveitis, BD, and sarcoidosis are the three main etiologies of non-infectious uveitis in western countries [1]. In this issue, El-Jammal et al. described three selected cases of these diseases, the patients being referred by ophthalmologists to their internal medicine department for unexplained uveitis [5]. In their tertiary referral center, 75 patients had been diagnosed with Spa (*n* = 20), BD (*n* = 9), or sarcoidosis (*n* = 46) in 2018 and 2019. There was a significant delay in the diagnosis of Spa-associated uveitis. Screening strategies using HLA-B27 determination and sacroiliac magnetic resonance imaging in patients suffering from chronic low back pain and/or psoriasis helped in the diagnosis [6]. Patients with BD’s uveitis were young people of both sexes and all ethnicities and usually presented with panuveitis and retinal vasculitis. El-Jammal et al. explained the high proportion of sarcoidosis in their population by the use of 18F-fluorodeoxyglucose positron emission tomography computed-tomography (PET-CT), which helped identify smaller hilar or mediastinal involvement, especially in the elderly [7].

Clinically isolated uveitis revealing sarcoidosis remained a strictly ocular condition in most cases. The occurrence of symptomatic extraocular involvement in patients with initially isolated sarcoid uveitis has been reported in 7.7% to 17% of cases [8,9]. Schupp et al. showed an association between eyes, cardiac, cutaneous, and CNS thanks to a clustering analysis [10]. However, the association between eye and cardiac involvement remained uncommon in this work (8.9%). In this issue, Richard et al. reported a retrospective cohort of 294 patients with sarcoid uveitis, in which only seven (2.4%) had cardiac sarcoidosis [11]. Interestingly, only 2 among 234 patients (0.85%) with sarcoid uveitis as a symptom developed cardiac sarcoidosis within five years of the presentation of uveitis. Conversely, cardiac sarcoidosis occurred more frequently in patients who already had symptomatic multisystem involvement or patients who progressed from an isolated ocular sarcoidosis to a systemic presentation.

A few studies have investigated the visual prognosis of sarcoid uveitis. Approximately 5% of patients have severe visual impairment [8,12,13,14]. The main cause of vision loss is cystoid macular edema [12]. Risk factors associated with poor functional prognosis according to studies are late-age onset, African-American origin, female sex, underlying chronic systemic sarcoidosis, posterior segment involvement, chronic cystoid macular edema, multifocal choroiditis, persistent ocular inflammation, and glaucoma [8,15,16,17]. In this Special Issue, Bienvenu et al. reported, for the first time, the factors associated with ocular and extra-ocular recovery in patients with sarcoid uveitis [18]. Recovery was defined by as a disease-free status, spontaneously or despite cessation of all treatments for three years or more. After a median follow-up of 83.5 months, recovery was reported in 37 of the 143 patients (26%). Caucasian ethnicity and anterior uveitis were significantly associated with recovery, whereas elevated intra-ocular pressure was negatively associated. Overall, this study showed that chronic sarcoid uveitis is a chronic condition and finds prognostic factors previously identified in systemic sarcoidosis.

BD represents 4.2% of all uveitis cases in the ULISSE study [19] and 1.8% to 6.1% of severe uveitis cases treated in European specialized centres [20]. On the other hand, the proportion of BD patients with ocular manifestations varies, according to studies, from 28% to 50% [21], with a higher incidence in young men [22]. Among patients with Behcet’s uveitis, 90% have posterior uveitis or panuveitis and 78% have bilateral involvement; furthermore, 89% have retinal vasculitis and 44% macular edema [23,24], which can lead to vision loss (with blindness in 13% to 21% of cases in series published in the last century) [23,24,25]. In this Special Issue, Gueudry et al. provided current data on how innovations in clinical evaluation, investigations, and treatments were able to improve the prognosis of uveitis associated with BD [26]. According to the 2018 EULAR recommendations for the management of BD, posterior segment involvement warrants systemic glucocorticoid therapy in combination with azathioprine or ciclosporin [27]. These treatments are considered as inadequate in patients with severe retinal vasculitis [28]. Acute sight-threatening uveitis and severe uveitis (e.g., severe inflammatory optic neuropathy, macular ischemia, unilateral uveitis in monophthalmic patients) are emergencies for BD patients. In these cases, methylprednisolone pulses (250–1000 mg/d for 1 to 3 days) are recommended [29]. The prognosis of severe or refractory forms has radically changed with the introduction of tumor necrosis factor-α (TNF-α) antagonists, mainly infliximab (IFX) [30,31,32]. These drugs are effective in 80% to 90% of cases, usually within a few days [31]. According to the 2018 EULAR recommendations, patients with an initial or recurrent episode of sight-threatening acute uveitis should be treated with IFX or interferon-α [27]. A recent study from the French Uveitis Network comparing IFX and adalimumab (ADA) in 330 sight-threatening uveitides, including 89 BD, showed that IFX was associated with a lower risk of relapse than ADA [33]. Interferon-α2a is also effective in posterior uveitis due to BD, with a two-to-four-week delay in its onset of action. In contrast to experience with TNF-α antagonists, interferon-α2a induces a sustained remission, which may persist after discontinuation of treatment (20% to 58% of cases) [34].

Juvenile idiopathic arthritis (JIA) is the most common rheumatic disease in children. Children with JIA are at risk for uveitis, which develops in approximately 12% to 38% of patients within seven years after the onset of arthritis [35,36]. In 2018, an expert group of the Single Hub and Access point for pediatric Rheumatology in Europe (SHARE) provided recommendations for the diagnosis and treatment of JIA-associated uveitis [35]. In his article, Pr Quartier offers a review on the recent therapeutic approaches in JIA-associated uveitis [37]. Two randomized, double-blind placebo-controlled trials, the large multicentre SYCAMORE trial in the United Kingdom and the smaller ADJUVITE trial in France, demonstrated the efficacy of ADA in patients with inadequate responses to local steroid therapy and methotrexate (MTX) [38,39]. A significant positive effect of ADA on uveitis was documented as early as two months in most patients using sensitive assessment tools such as laser flare photometry [39]. A multicentre, single-arm phase II trial, APTITUDE, recently assessed the safety and efficacy of tocilizumab in children with JIA-associated uveitis refractory to both MTX and ADA [40]. Although the primary endpoint of the trial was not met, as only 7 out of 21 patients achieved a significant improvement of anterior chamber inflammation according to the SUN criteria [41] after 12 weeks, the authors considered that tocilizumab was beneficial in a subset of patients; in particular, macular edema was present in four patients at baseline and resolved in three cases. This treatment is certainly interesting, as only hard-to-treat patients were included in this trial, as their uveitis was refractory to MTX and ADA therapy. Baracitinib, an oral JAK-1/2 inhibitor is being tested in patients with antinuclear antibodies-positive, early-onset, idiopathic or JIA-associated uveitis in an ongoing international trial [42] and may add the therapeutic armamentarium for treating this condition.

Birdshot chorioretinopathy (BCR) is a non-infectious, bilateral, uveitis predominantly affecting the posterior segment with independent retinal and choroidal dual inflammation and almost exclusively seen in Caucasians [43]. Studies in Europe and in the USA showed that BCR accounts for 0.5% to 1.5% of all uveitis seen in specialist uveitis practices and around 10% of posterior uveitis [44]. The association of BCR with the HLA-A29 major tissue histocompatibility antigen is estimated to be close to 100% [45], while this antigen is present in around 7% of Caucasian populations [43]. In this issue, Bousquet et al. [46] provided a review on BCR with a focus on choroidal imaging techniques to assess disease activity and monitor its progression. Retinal vasculitis is diagnosed and monitored with fluorescein angiography with findings such as retinal vascular leakage, nonperfusion, and neovascularization, while indocyanine green angiography is essential in detecting occult choroiditis and perform early diagnosis [47]. Optical coherence tomography (OCT), in particular the spectral domain (SD)-OCT (SD-OCT), is an excellent method to detect and monitor macular edema, which is reported in one third of the patients [48]. Secondary development of enhanced depth imaging SD-OCT allowed the characterisation of choroidal thickness and choroidal reflectivity changes in active and inactive BCR [49]. Fundus autofluorescence provides insights into the metabolic state of the photoreceptors/retinal pigment epithelium based on the presence of lipofuscin and could also be useful in evaluating disease activity and guiding follow-up and treatment [50]. OCT angiography is a new imaging modality that assesses the perivascular thickness of large-vessels, which can be used for monitoring posterior pole vasculitis and potentially the progression of the disease [51,52].

Finally, Chaudot et al. reported on ocular immunotherapy-related adverse events (IRAEs), which are newcomers to the field of ocular inflammation [53]. IRAEs again illustrate the importance of collaboration between ophthalmologists and other medical specialists, here oncologists and internists. Currently, less than ten immune checkpoint inhibitors (ICPIs) are approved by the US FDA and the European Medicines Agency [54], but many ICPIs are still in development. Collecting the cases of 28 patients (corresponding to 36 ocular IRAEs), they found that the mean time from ICPI introduction to ocular IRAE was 17 weeks (±19). Most patients were treated for metastatic melanoma, as this was the first indication for ICPI. Anti-PD1 agents (i.e., the “older” agents) were responsible for the majority of IRAEs. Uveitis was mainly anterior and bilateral. Interestingly, two-thirds were granulomatous. There were also rare cases of Vogt–Koyanagi–Harada-like or birdshot-like syndromes. Although most ocular IRAEs were considered severe (70% grade 3), they were easily controlled by local or systemic corticosteroids. Notably, ICPI was discontinued in 60% of cases. This study was accompanied by an extensive literature review of 230 cases of uveitis. The results of this huge bibliographic work were similar to those of their series, except for a much lower frequency (7%) of granulomatous features. In addition, discontinuation of ICPI did not appear to alter the prognosis of ocular IRAEs. With the increasing use of ICPIs and the growing number of reports, the optimal management of ocular IRAEs should soon be better defined.

In conclusion, while not all of the numerous questions raised by non-infectious uveitis could be included in the register of this Special Issue, such as, for example, the potential role of the microbiota [55] or the value of measuring cytokine levels in aqueous humor, tears, or serum to help the diagnosis of specific inflammatory disorders and stratify patients for tailored treatments [56,57,58,59], we believe that readers will find significant information herein for their research or daily practice.
